# A Common Anterior Insula Representation of Disgust Observation, Experience and Imagination Shows Divergent Functional Connectivity Pathways

**DOI:** 10.1371/journal.pone.0002939

**Published:** 2008-08-13

**Authors:** Mbemba Jabbi, Jojanneke Bastiaansen, Christian Keysers

**Affiliations:** 1 Section on Integrative Neuroimaging, Cognitive Brain Disorders Branch, National Institutes of Mental Health, Bethesda, Maryland, United States of America; 2 Social Brain Lab, BCN Neuroimaging Center, University Medical Center Groningen, Groningen, The Netherlands; Victoria University of Wellington, New Zealand

## Abstract

Similar brain regions are involved when we imagine, observe and execute an action. Is the same true for emotions? Here, the same subjects were scanned while they (a) experience, (b) view someone else experiencing and (c) imagine experiencing gustatory emotions (through script-driven imagery). Capitalizing on the fact that disgust is repeatedly inducible within the scanner environment, we scanned the same participants while they (a) view actors taste the content of a cup and look disgusted (b) tasted unpleasant bitter liquids to induce disgust, and (c) read and imagine scenarios involving disgust and their neutral counterparts. To reduce habituation, we inter-mixed trials of positive emotions in all three scanning experiments. We found voxels in the anterior Insula and adjacent frontal operculum to be involved in all three modalities of disgust, suggesting that simulation in the context of social perception and mental imagery of disgust share a common neural substrates. Using effective connectivity, this shared region however was found to be embedded in distinct functional circuits during the three modalities, suggesting why observing, imagining and experiencing an emotion feels so different.

## Introduction

“Disgust refers to something revolting, primarily in relation to the sense of taste, as actually perceived or vividly imagined; and secondarily to anything which causes a similar feeling, through the sense of smell, touch and even eyesight” Charles Darwin (1872/1965)

The concept of ‘simulation’ is important for our understanding of imagination and social perception. For actions, simulation accounts of *imagination* propose that we can accurately imagine what it feels like to perform actions because common brain areas are involved in the execution and imagination of these actions. Empirical evidence showing parietal, pre-/supplementary motor cortex activations during imagination and action-execution supports this account [1]–[5]. Simulation accounts of action *perception* posit that we intuitively feel what others do and can anticipate their future actions because our perceptual apparatus links their actions with neural structures planning our own actions. Empirical support for this comes from the discovery of mirror neurons in the premotor and inferior parietal cortex of the macaque monkey responding to perception and execution of similar actions [6]–[9] and the observation of human premotor and inferior parietal responses to observation and execution of actions [10]–[16].

Together, evidence for action simulation, perception and imagination implicates brain areas including the premotor and posterior parietal regions as neural substrates involved in three functions: motor execution, observation and imagination. Perhaps the brain does not need to duplicate the motor expertise stored in motor areas in order to permit imagination and social perceptive processes: at least in part, it employs the very hardware of our own actions. However, it is unclear whether these notions can be extended to the realm of emotions.

Neuroimaging studies have shown that while individuals view or become aware of the delight [17], pain [18]–[21] or disgust [17], [22], [23] of others, they activate the anterior insula and adjacent frontal operculum (IFO) that reacts to experience of similar emotions and is modulated by empathic tendencies. IFO lesions also disrupt experience and recognition of disgust, suggesting a role for this region in emotional simulation/understanding [24], [25].

Interestingly, similar IFO regions have been shown to be recruited during affective autobiographical recall [26], [27], and taste imagination of pictured food items [28]. In line with these results, emerging evidence points to the functional significance of this region in facilitating awareness per se [29]. Thus in addition to deficits in disgust perception and experience, lesions of IFO has been shown to result in marked reduction of feelings of craving for cigarettes in long term smokers [30], [31], anosognosia [32] and amusia [33].

Given the well documented role of the IFO in coding experience and social observation of disgust (among other feeling states), an interesting question would be whether this area would similarly respond to individuals vivid imagination of disgusting experiences. Here, we address this question and further examined how the functional circuitry that includes the IFO differs between the imagination, observation and experience of disgust. In with the emerging role of this region in coding awareness of feeling states, it was hypothesized that a common IFO region involved in the experience and observation of disgust [17], [23], will be involved in imagination and that the functional connectivity between this region and the rest of the brain will differ across experience, observation and imagination of disgust.

## Materials and Methods

*Participants:* 12 healthy right-handed volunteers (6 females) were recruited for the three fMRI experiments. All 12 individuals were free of neurological, psychiatric and other physical conditions with normal or corrected to normal vision. They completed the consent forms approved by the University Medical Center Groningen's institutional review board and were paid 50 euros in total for their participation to *observation*, *experience* and *imagination* experiments.

*Observation and Experience:* The experimental procedures for the observation and experience conditions of this study have been described earlier [17], but are briefly illustrated in Figures S2 and S3.

*Imagination:* Similar to the observation and experience experiments, the script-driven imagery runs consisted of three different conditions: disgust, neutral and pleasant (but the data of the pleasant condition is not of interest to the present report). Written scenarios (scripts) with an approximate reading time of 35 seconds were developed to induce disgust (9 scripts), neutral (7 scripts) and pleasant (8 scripts) emotional feeling states, totaling to 25 scripts (see Supplementary Materials S1 for sample scripts).

Initially, 11 participants that did not take part in the fMRI study rate all 25 scripts as to the amount of disgust and pleasure they experience while reading and imagining themselves going through the scenarios of the scripts and how hard the scripts were to imagine. We asked two additional participants to tell us which script they read most quickly, and then measured their reading time with a chronometer. The shortest reading time was just over 20 s, which is why 20 s was used as the upper limit of the surface under the curve analysis described below (For additional information, see Supplementary Materials).

For the final experiment, each of the participants rated all 25 scripts in terms of how disgusting, how pleasant and how hard to imagine (on a scale ranging from 0 to 6). These ratings served two purposes: first, to choose on an individual basis the 6 most disgusting, the 6 most pleasant and the 6 most neutral (i.e. least disgusting or pleasant) scripts for the fMRI experiment, and second, to obtained personal ratings of the scripts used in the fMRI experiment (Figure S1).

During scanning, (Figure S4) each trial begins with a red fixation cross lasting 6 seconds followed by a script present as a single screen of text lasting 35 seconds followed by a fixation cross lasting 6 seconds. The subjects then viewed a screen with a simple arithmetic task for 6 seconds, and had to indicate their choice by pressing the right or left button of a response box with their right index finger. The next trial then begun with 6 seconds of fixation cross and the next script and so on. We included the arithmetic task between two scripts to maintain attention and wash out the emotional state induced by the scripts between two scripts. Each run contained 9 trials (3 disgust, 3 neutral and three pleasant scripts, all presented in a fully randomized order) and two such runs lasting 9.35 minutes each were administered for the imagination experiment.

*Image Acquisition and Analysis:* Images were acquired using a Philips 3T whole-body scanner (Best, The Netherlands) using a circular sense head coil. T2*-weighted echo-planar sequencing was performed with 39 interleaved 3.5 mm thick axial slices with 0 mm gap (TR = 2000 ms, TE = 30 ms, flip angle = 80°, FOV = 224 mm, 64×64 matrix of 3.5×3.5×3.5 mm voxels). At the end of each functional scan, a T1-weighted anatomical image (1×1×1 mm) parallel to the bicommissural plane, covering the whole brain was acquired.

Statistical Parametric Mapping (SPM2; Wellcome Department of Cognitive Neurology, London UK; http://www.fil.oin.ucl.ac.uk) was used for the preprocessing and analysis. All functional volumes were realigned to the first acquired volume and images were then coregistered to the participant's anatomical space and subsequently spatially normalized to obtain images with a voxel size of 2×2×2 mm [34]. All volumes were then smoothed with an 8 mm full-with half-maximum isotropic Gaussian kernel. For the time series on all 12 participants, high-pass filters with cut-off points at 106 s, 310 s and 380 s for the observation, tasting and imagination conditions respectively, were included in the filtering matrix in order to remove low-frequency noise and slow-drifts in the signal. Condition-specific effects at each voxel were estimated using the general linear model. Contrast images were then tested at the group level using a one tailed t-test against zero to implement a random effects analysis. We extracted the timecourse from the IFO ROI that was found to be commonly active during the observation and experience [17] for all three experimental conditions using marsbar (http://marsbar.scourceforge.net; M.Brett, J.-L. Anton, R. Valabregue, and J.-B. Poline, ROI SPM toolbox, Abstract).

*Connectivity Analysis:* To explore the functional integration between the shared circuit mechanism in the IFO and other related regions during the three disgust modalities, we employed the psychophysiological interaction (PPI) analysis implemented in SPM to identify voxels whose timecourse correlated more strongly with the timecourse of activity in IFO during the disgust compared to the neutral condition for imagination, observation and taste separately using the procedures advised by Friston and colleagues [35]. The seed region for this analysis was determined in each condition and subject separately by opening the relevant contrast in SPM (e.g. vision of disgust – vision of neutral), placing the cursor at the center of the IFO ROI (x = 42, y = 18, z = −6) and defining a 5 mm radius sphere using the function VOI. This function will automatically move to the closest voxel with a significant contrast (at p<0.005 uncorrected), and the actual center of the sphere therefore deviated on average by 4 mm from the center of the ROI (see Table S2), but their was no significant difference between the spatial distribution of centers in the three modalities (two-tailed matched pair t-test performed separately on the x, y and z coordinates for imagination vs. observation, imagination vs. taste and observation vs. taste, all p>0.25 uncorrected). The PPI analysis multiplies point by point the timecourse of activity in the sphere seed region with a psychological variable containing the value 1 for the condition disgust, −1 for the condition neutral and zero elsewhere and then uses this interaction vector, next to both the timecourse of the seed region and the psychological variable as three regressors in a subsequent whole-brain GLM analysis. Comparing the parameter estimate of the interaction term with zero at the second level of analysis (one-tailed t-test comparing n = 12 parameter estimates against zero) then identifies voxels in the population of 12 participants that are on average functionally more connected to the seed region in the disgust condition compared to the neutral condition (Friston et al., 1997). These PPI maps were then thresholded at p<0.001, uncorrected, with an extent threshold of 10 voxels.

## Results

### Script ratings

Before scanning, the twelve participants included in the fMRI experiment rated (Figure S1) the 6 disgust scripts as more disgusting than the neutral ones (two-tailed matched pair t-test, p<0.001 uncorrected for multiple comparisons) but their was no significant difference between the disgusting and neutral scripts in terms of how pleasant (p>0.07) or how hard they were to imagine (p>0.87). The pleasant scripts that served to balance the experimental design but that were not further analyzed here differed from the other scripts in that they were less disgusting than the disgust script (p<0.001), more pleasant than both the other types of scripts (p<0.001). Finally, the disgusting scripts were slightly more disgusting than the pleasant scripts pleasant (p<0.04).

### Timecourses

**Figure 1:**
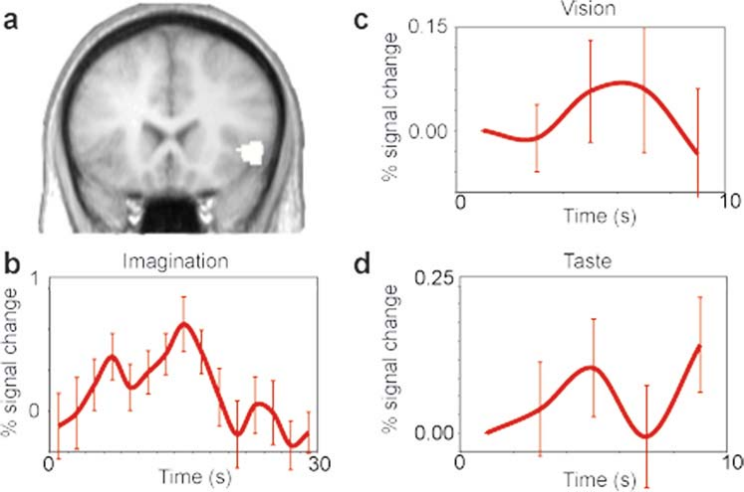
Condition specific IFO time courses. a) coronal slice (y = 18) showing the location of the ROI (white) previously shown to be involved in the experience and observation of disgust (p<0.005, k>10 voxels) (Jabbi et al., 2007). b–d) time courses of the average disgust-neutral difference relative to the onset of the movies of facial expressions, script-driven imagery and the administration of the tastants, respectively. Error bars represent the standard error of the mean.

In Jabbi et al. [17], a region of the IFO was significantly more active during the vision and the experience of disgust compared to their neutral control conditions (p<0.005 vision of disgust – vision of neutral and p<0.005 taste of quinine – taste of neutral solution, Fig. 1a). To examine if this region is also recruited during the imagination of scenarios involving disgust (compared to those without emotional valence) we extracted the signal from this ROI in the imagination condition for the 12 participants that returned to be scanned during the imagination of emotional scripts (Fig. 1b; Traditional GLM results for all three modalities are specified in Table S1). Given that it is difficult to know how the emotional state of the participants fluctuates during the reading of the scenarios, we did not use a standard GLM approach but instead calculated the surface under the average difference curve between the disgust and neutral scenarios for the interval 4 s–20 s for each individual. The first 4 s were excluded because of the hemodynamic response delay and time points after 20 s, to exclude volumes in which some of the participants had finished reading some of the scripts. One of the core goals of the present study being the examination of a shared IFO representation of the simulation (imagination), experience and social observation of emotional feeling states, we therefore employed a one-sample t-test (one tailed) comparing the surfaces of the 12 participants against zero and found the disgust scenarios significantly (p<0.004) recruit the IFO ROI more than neutral scenarios during imagination. Timecourses of the IFO ROI during the observation and experience of disgust are also shown for illustrative purposes [17], [23].

### Functional Connectivity

**Figure 2:**
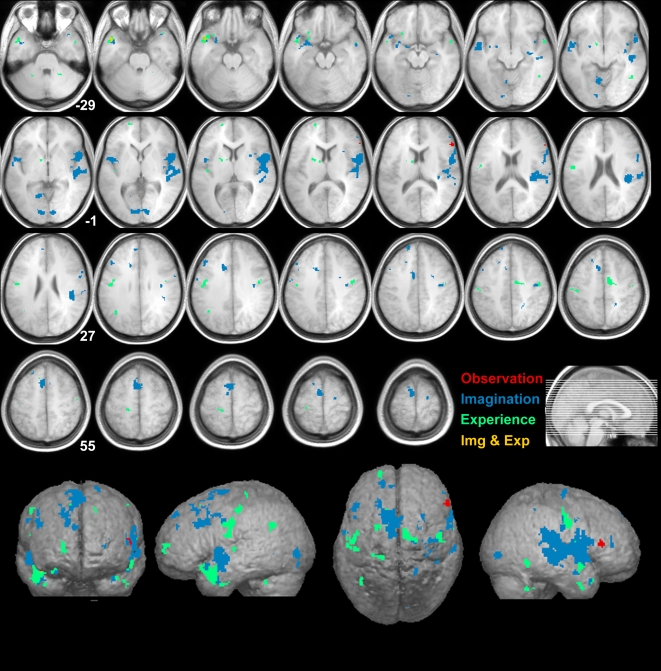
PPI maps of the whole brain. Functional connectivity of the whole brain with the IFO (as seed) thresholded at the t = 4.64 (corresponding to p<0.05 corrected for false discovery rate for the imagination of disgust relative to neutral). The numbers in the figures shows the corresponding Z-coordinates in MNI space. Left is left and right is right.

To examine the functional circuitry within which the IFO is embedded in these three disgust modalities (observation, imagination and experience), we used the time course of the IFO (based on a 5 mm sphere centered on the voxel with a significant omnibus test closest to x = 42, y = 18, z = −6, see methods) as the seed region to map effective connectivity using three separate (one per modality) psychophysiological interaction (PPI) analysis [35]. This analysis was performed separately for each participant, and the parameter estimates of the interaction term tested against zero at the population level using a one-tailed t-test to determine which voxels consistently increased their functional connectivity with the IFO during the disgust condition compared to the neutral condition. Results are shown in Figure 2 and Table 1.

**Table 1:** Regions of Effective Connectivity with IFO during Observation, Taste and Imagination of Disgust Relative to Neutral at p<0.001, k>10 voxels, t>4.64.

Observation PPI						
*Region*	MNI			Voxels	t-value	z-value
	X	Y	Z			
IFG BA45	56	30	12	26	6.35	4.04

Taste PPI						
*Region*	MNI			Voxels	t-value	z-value
	X	Y	Z			
Temporal Pole	−48	5	−21	147	7.98	4.5
SI/SII	−50	−14	31	134	8.32	4.59
	−55	−23	51	12	5.51	3.74
Cingulate Motor	11	−12	52	89	7.39	4.35
MI BA/3a/4a/4p	46	−13	43	60	7.02	4.24
	−20	−35	64	32	5.83	3.86
	−46	−27	53	17	5.78	3.84
Orbitofrontal Cortex	−28	12	−15	37	6.67	4.14
MPFG	−19	57	9	31	7.14	4.28
Putamen/Lentiform	−22	−2	12	30	5.18	3.61
Caudate	−12	−5	14	14	4.64	3.3
Globus pallidus	−13	−2	−2	14	5.9	3.88
Angular gyrus/TPJ	−40	−62	33	28	5.76	3.83
ITG/MTG	56	2	−9	23	5.62	3.78
	49	2	−30	12	4.98	3.53
ITG	46	−58	−5	21	6.31	4.02
	−39	−8	−31	18	5.57	3.76
Lingual Gyrus	−26	−67	−5	10	5.33	3.67
ITG/MTG/STG	−38	−4	−36	18	5.57	3.76
	50	8	−38	12	4.98	3.53
Posterior Insula	−45	−20	8	11	5.09	3.53
Cerebellum	41	−58	−34	12	4.98	3.53
	20	−52	−28	19	8.5	4.63
	−24	−55	−29	14	5.41	3.7

Imagination PPI						
*Region*	MNI			Voxels	t-value	z-value
	X	Y	Z			
Mid&Post Insula/STG	50	8	−4	1754	12.51	5.38
Lingual Gyrus	−4	−90	2	202	5.19	3.62
	−11	−64	−1	102	6.11	3.96
Temporal Pole/MTG	−46	4	−20	129	7.3	4.32
	36	13	−25	10	5.99	3.91
	52	−3	−16	13	5.16	3.61
SMA/preSMA BA6	−6	3	63	374	6.02	3.92
Amygdala	−31	−2	−13	129	7.3	4.32
Hyppocampus	34	−16	−4	56	6.5	4.08
MI	19	−17	73	28	7.42	4.36
ACC	−15	12	37	57	7.24	4.3
IFG BA44/45	−47	20	36	44	4.84	3.47
Posterior cingulate	−8	0	45	33	4.99	3.54
Prefrontal gyrus/SFG	−17	46	46	31	4.9	3.5
	−21	24	51	24	5.33	3.67
Premotor cortex/BA6	38	−17	42	67	5.05	3.56
	−59	−8	39	10	4.64	3.38
MPFG	−7	44	32	13	5.17	3.61
Putamen	30	8	10	11	5.19	3.62

During observation, we found only the ipsilateral right inferior frontal gyrus (IFG, pars triangularis or BA 45 [36] to be effectively more connected with the right IFO during the observation of facial expressions of disgust relative to neutral faces (Table 1).

During experience and imagination condition, a much wider network involving in particular somatosensory, motor, gustatory and ‘limbic’ regions were shown to be effectively more connected to the IFO during the disgust compared to the neutral condition (Table 1).

Overlap between the functional connectivity networks observed in these modalities was rare and restricted only to a single two-way overlapping cluster between imagination and experience in the left temporal pole.

## Discussion

We tested and confirmed the hypothesis that the shared-circuitry in the IFO, shown earlier to be active during experience and observation of other people's disgust [17], [23], is also activated by the imagination of one's own disgust.

The IFO is thought to play an important role in interoceptive awareness, i.e. sensing the inner state of the body [30], [31], [37]–[39]. This regions' involvement in both the observation and experience of disgust and pleasure [17], [23] has been speculated to provide a simulation mechanism of the inner state of disgust during disgust observation in others. Findings showing IFO involvement in experience and observation/awareness of other people's pain [18]–[21], suggested that this purported simulatory IFO response may not be specific for emotions linked to gustatory or olfactory stimulation but rather more generally linked to the simulation of bodily feeling states during social cognition [40]–[41]. Independent evidence suggests that some sectors of IFO are also involved in the imagination of basic emotions and sensations such as taste [26]–[28].

Our findings of a common IFO activation in the same participants' experience, observation and while they imagine feeling disgust provides to our knowledge, the first direct evidence indicating that two apparently distinct forms of simulation (social perception and imagination) actually share a common neural substrate in the IFO. These findings have two implications: First, it supports the idea that imagination and social perception of emotions may share neuroanatomical underpinnings. This is in line with similar findings in the mirror neuron literature showing common neural representations for perceived, executed and imagined motor actions [6]–[9]. Second, it provides insights into the neural basis of the captivating experience of reading a book: While previous studies on social perception used movies of other people's experiences or arbitrarily colored symbolic cues, our combination of movies and written material in the present experiment demonstrates that reading (mental imagery) as well as watching other people experience what is imagined recruits brain regions involved in experiencing an emotion.

The IFO ROI selected in this study appears to be a key location in the phenomenon of simulation that makes feeling an emotion, seeing that emotion on someone else's face and imagining that emotion somehow shares a similar feeling component. Despite this partial overlap, these three modalities of disgust however do feel clearly different, emerge through distinct processes, and are triggered by different events. During experience: the brain activity is triggered by an unpleasant taste; during observation: by the sight of disgusted facial expression; and during imagination: by vivid mental imagery triggered by written scripts. Interestingly, these differences seem to be reflected in our connectivity findings.

Our IFO ROI involved in all three modalities includes anterior aspects of the insula and the adjacent frontal operculum, where the postmortem cytoarchitectonic analysis of 5 human brains observed a dysgranular cytoarchitecture [42], probably corresponding to the dysgranular zone of the frontal operculum/insula [43]. Tracer injections in the monkey Insula shows it to be highly interconnected with most of the brain [44]–[46], in particular the motor cortices (IFG-premotor, SMA/preSMA, M1 and cingulated motor cortex), regions involved in gustation (basal ganglia, amygdala, ACC, orbitofrontal cortex), somatosensation (SI, SII and posterior Insula), high level vision (STS) and memory and semantics (temporal pole and hippocampus). However, it is worth noting that the monkey Insula does not have a homologue of IFO [47], underscoring a likely prominent role for this phylogenetically new region in higher order physiological awareness “that might be absent in monkeys”. Our effective connectivity findings showed changes in the temporal correlation between the BOLD signal in the IFO and a variety of putative human homologues of the connected structures shown earlier to be connected with monkey insula [43]–[46].

During experience, changes in effective connectivity occur primarily with somatosensory (left SI/SII and posterior Insula) [48], gustatory/ motivational (basal ganglia, orbitofrontal cortex), and motor output regions (cingulated and primary motor cortex). What do these changes in effective connectivity mean? The SI/SII and the posterior insula are involved in somatosensation [49] and could represent the tactile experience of tasted fluids - relatively similar for neutral and unpleasant gustatory stimuli. The IFO however increases activity as the intensity of the taste of a solution increases as well as integrates the taste and texture of food [49]. The observed IFO effective connectivity with somatosensory areas during disgust experience relative to tasteless artificial saliva may therefore likely reflect the integration of texture and taste in the IFO. Indeed, the orbitofrontal cortex, the basal ganglia and motor regions (M1 and cingulate motor cortex) are involved in evaluating the valence of a taste [49] and regulating behavior accordingly [47]. Thus the increase in effective connectivity between these regions and the IFO may underlie the valence-related relevance of taste processing.

During imagination, participants need to (a) transform the written material involved in the scripts into a mental simulation of the actions, sensations and feeling states of the protagonists. All scripts, be they disgust-inducing or neutral involved actions and sensations, and this processing would therefore not be specific for the disgust inducing scripts. Unlike the neutral scripts, imagining the disgust inducing scripts naturally triggers strong feeling states of disgust. Broca's area (left BA44/45) and the left temporal pole are structures that are known to be important for understanding stories [50]. Thus, the increase of effective connectivity between the IFO and these regions for the disgust scripts may likely reflect a cognitive-affective integration mechanism. The SMA/preSMA plays a key role in the mental imagery of actions [−5, 51] and somatosensory regions (right SI/SII/posterior Insula) play an important role in the mental imagery of tactile and proprioceptive sensation [48], [51] and would therefore play an important role in the imagery of actions and sensations in general. The change of effective connectivity with the IFO however reflects that this motor and somatosensory imagery seems to be linked to activity in the IFO and feeling states more strongly, if these actions and sensations are disgusting. Increases of connectivity with the hippocampus finally could reflect autobiographic memories triggered by the scripts [52].

During social observation, the most prominent region with stronger connectivity during disgust compared to neutral faces was the ipsilateral right BA45. This region has been shown to be involved in execution, observation and imitation of facial expressions [22], [53], [54]. Together, these findings suggest that vision of any facial movement triggers a motor simulation of facial expressions in the BA45 that might be related to the phenomenon of facial mimicry [55], [56]. If the facial expression is emotional however, and disgusting in particular, an increase in effective connectivity between this region and the IFO, would link a simulation of the bodily feeling state of disgust with the simulation of the disgusted facial expression. Indeed, whereas lesions of the IFG resulted in widespread deficits in the perception of facial expressions [57], lesions in the IFO lead to more focused deficits in disgust recognition [24], [25].

### Conclusions

Humans can achieve vivid emotional feeling states in the absence of actual emotional encounters in a myriad of ways, including the recall of past experiences, the imagination of hypothetical experiences, reading a good book, watching a good movie or witnessing a friend's experience. By making participants view disgusted facial expression of others, read disgust provoking scenarios and taste an unpleasantly bitter solution, we found a modality *a-specific* involvement of a region of the IFO during disgust. However, the functional connectivity between this region and the rest of the brain was orchestrated in a modality *specific* way. This suggests that the IFO is a convergence zone where bodily feeling states relevant for the emotion of disgust are coded according to a common code [58], [59] regardless of stimulus modality. Our findings of IFO involvement in all three modalities supports the idea that simulation through both pre-reflective (viewing someone else's disgust) as well as reflective (deliberate mental imagery and language) routes may therefore be complementary rather than independent of each other [60]. This idea is supported by evidence showing dampening effect of people's expectation on their IFO response during exposure to aversive tastes, suggesting a role for this region in regulating reflective/cognitive processes relevant for homeostatic maintenance [61]. The functional relationship between the IFO and interconnected regions during social cognition, as opposed to imaginary and actual emotional experience remains an important question for future work, but the relative lack of overlap between the results of our effective connectivity analysis between the three modalities confirms the idea that these modalities feel different despite the presence of regions that encode them according to a common code because they are embedded in distinct, and modality specific neural circuitries [59], [60]. In sum, our findings of IFO involvement in the actual imagination of gustatory disgust are in support of the important role of this region in regulating awareness and embodiment of feeling states.

## Supporting Information

Supplementary Materials S1 (0.04 MB DOC)

Figure S1 Script rating. The 12 participants of the fMRI experiment rated all 25 available scripts on a scale ranging from 0–6 according to how disgusting, how pleasant and how hard to imagine they find them. On an individual basis, the 6 most disgusting, the six most pleasant and the six most neutral (i.e. least disgusting and least pleasant) scripts were then chosen for inclusion in the fMRI experiment, and the average rating of the chosen scripts shown in this figure (error bars representing the standard error of the mean over the 12 subjects). * denote significant matched-pair t-tests (2 tailed, p<0.01 uncorrected). Note that ratings were only compared within each rating (i.e. the three scripts were compared separately in terms of how disgusting they were, how pleasant they were and how hard they were to imagine).
(5.18 MB PDF)

Figure S2 Frames represent different time points of the 3 s movies depicting facial expressions of disgust, neutral and pleased gustatory experiences. See Jabbi et al., 2007 for detailed description of this part of the methods.
(1.30 MB PDF)

Figure S3 Sequence of events within a single taste trial. The person with the headphone represents an experimenter while the individual lying supine represents a participant in the scanner with three tubes protruding into a pacifier in the mouth through which various tastants are delivered. See Jabbi et al. for detailed description of this part of the methods.
(0.47 MB PDF)

Figure S4 Structure of an imagination trial in the scanner.
(1.65 MB PDF)

Table S1 (0.10 MB DOC)

Table S2 (0.05 MB DOC)
