# Supplementary material for: A Common Anterior Insula Representation of Disgust Observation, Experience and Imagination Shows Divergent Functional Connectivity Pathways
Source: PLoS One. 2008 Aug 13;3(8):e2939. doi: 10.1371/journal.pone.0002939 (PMC2491556; doi:10.1371/journal.pone.0002939)
Supplement: Supplementary Materials S1 — (0.04 MB DOC) [file pone.0002939.s001.doc]

**SUPPLEMENTARY MATERIALS**

**Supplementary Notes**

**Sample Scripts**

The original scripts are in Dutch language. Below you will find 4 examples of each category translated in English.

**Disgust**

**1.** You are sleeping for a night in a decrepit inn in the Czech Republic. When you park yourself on the bed, you see the decomposing head of a rat rocking with your movement on the mattress. In a reflex you smack the cadaver off the sheets with your bare hand. To prevent yourself from gagging your hand reaches for your mouth. But when your hand makes contact with your face, you become even more nauseated. Your fingers are covered with a red gunk, covered with swarming maggots! You feel the tacky substance stick to your cheeks and lips. Finally, the horrid taste of tainted blood enters your mouth…. (108 words)

**2.** You are a guest in the cottage of an acquaintance of a good friend of yours who own an uncommonly clingy cat. When you step out of the shower in the morning, you bury your wet face in a towel. A lukewarm, gooey matter encloses your face while a penetrating smell enters your nostrils. When you look in the towel, you see it is covered in the light brown lumpy crap of a cat. It is not until then that you sense the abysmal taste of feces on your lips. Between your teeth you feel a bone that once was part of the cat food. As you try to spit the bone out you can feel the feces spread through your mouth…. (122 words)

**3.** You are at a party where they are serving a true traditional delicacy. Round, slippery cows eyes covered in tiny red blood vessels are laying on plates. A feeling of horror runs right through your body. To be polite, you decide to take a bite. When you finally manage to get the slippery ball in between your jaws, it completely bursts inside your mouth. A tough membrane stays behind in your mouth, while the aqueous humor seeps out of the corners of your mouth. It is so horrendous that no matter how hard you try to swallow the membrane, your body keeps opposing it. Finally, you feel gastric acid coming from your stomach up to your throat.… (117 words)

**4.** When you turn around to look who is leaning on your shoulder, you are peering into the unsightly face of a homeless guy. When you try to free yourself from his penetrating presence, you briefly see a glance of his rotten teeth that are circled with bad sores before his eyes start to roll. The guy leans forward and discharges the complete content of his inflamed stomach on you! You are covered with decaying vomit that was formed by rotten meat picked out of the garbage cans across the street. It has been a long time since you have felt this sick. You can feel your stomach contract. Then you feel a hard, fleshy piece of his vomit in the corner of your mouth…. (124 words)

**Pleasant**

**1.** It is a rainy day and you have spent the whole day inside wearing a warm sweater. In front of you there is now a big, steaming pan on the table. The delicious, spicy smell of traditional soup spreads across the room. You are handed a full bowl of soup and snuggle yourself into the couch. The soup is very clear and generously filled with vegetables, vermicelli and crispy croutons. Once you have brought the spoon to your mouth, you taste the delightful combination of slightly salted soup with soft vermicelli and fresh vegetables. You feel how a warm glow runs through your body, while the slightly salty taste caresses your palate… (112 words)

**2.** Together with a friend you enter the woods for a nice long winter walk. There is a fair amount of snow, but you are warmly dressed. After an hour’s walk, the two of you slide in front of the fireplace in a cozy café. You order a cup of hot chocolate with cream. You see the steam coming off the cup, and with a biscuit you catch the cream that is melting together with the chocolate and then take a bite. A sense of well-being runs as a light shiver down your spine. The whipped cream is so full of taste and the biscuit is so wonderfully sweet. With your eyes closed you enjoy this tongue tingling experience… (118 words)

**3.** You join a friend at a family party that climaxed with a big buffet. By the time all the speeches are finished, you are very hungry. Your eyes wander around all the dishes with delicacies and it makes your mouth water. You gather a variety of the most enticing foods on your plate and sit yourself down. The food looks delicious and when you take a bite of a tasty seasoned piece of chicken and experience how tender the meat is, you are sure you will feast upon this meal. While you are still enjoying the tingling taste of juicy meat with fresh herbs, you feel how your body relaxes completely… (114 words)

**4.** The strawberry season just started again. You did not realize how good the strawberries were this season until you visit the marketplace one morning and see an abundance of fresh and juicy strawberries shining in the sunlight. You decide to have your share of the succulent fruit and cannot resist the temptation of secretively biting into one of them. As you inhale the sweet scent of the strawberry while opening your mouth for a bite, your lips touch its soft surface and without any hesitation your teeth go through it releasing the delicious sugary taste of one of the best strawberries you have had in a long time. You keep your eyes closed as if to avoid all distractions as you take the last bite of the strawberry. You feel your taste buds tingle as the strawberry melts on your tongue… (141 words)

**Neutral**

**1.** You are sitting behind your desk filling out some papers. In front of you there is a long, yellow pencil with black stripes widthwise. Careless you take the pencil from your desktop. The tip is sharpened recently and feels smooth. When you roll the pencil between your thumb and index finger, you feel that it has six even surfaces. At the other end the pencil is flat and a bit rough. When you turn your gaze back to the papers, you softly chew on the end of the pencil. You can taste the difference between the wood and the lead. With your tongue you explore the soft wood that is in between your teeth, but it hardly tastes like anything and leaves your taste buds cold… (126 words)

**2.** You just came back from grocery shopping and have your hands full with all kinds of things. While you try to hold all the bags with one hand, the other one searches for the keys of your front door. You come across a smooth, plastic pass in your coat pocket en feel the relief of the letters on it. When you take it out of your pocket it turns out to be a pass for the supermarket. You clasp the blue object in between your lips. Your tongue pushes against the blunt brim of the tasteless, plastic pass. When you put your bags down, you take the pass out of your mouth again… (113 words)

**3.** When you turn on the shower in the morning, a fair spurt of water clatters from the shower head to the floor. The water is clear and feels reasonably warm. Like a drizzle you feel the water fall on your shoulders and back. When you wet your hair, you let a small amount of water run into your mouth. After a while the water on your tongue feels the same as it does on the rest of your body. You are indifferent to the odorless fluid as it runs through your mouth. Then you let the water run down with the rest of the stream again… (106 words)

**4.** You promised to help a friend arrange a set of letters to make sure they are all sent out on time. When you come in, there are lots and lots of long white envelopes packed in a wooden box on a table. When you pick up one of the envelopes, you feel the slippery surface gliding through your fingers. You realize that instead of being straight, the edges of the envelope are instead cut in a sort of indented fashion. Without much concentration you take one envelope to your lips and lick the sticky part of the opening to make sure your saliva gets the line wet. You can feel the tastelessness of the glue-like substance. You close the envelope and repeat this procedure many times by licking and closing each envelop… (131 words)

Supplementary Figure Legends

**Figure S1. Script rating.** The 12 participants of the fMRI experiment rated all 25 available scripts on a scale ranging from 0-6 according to how disgusting, how pleasant and how hard to imagine they find them. On an individual basis, the 6 most disgusting, the six most pleasant and the six most neutral (i.e. least disgusting and least pleasant) scripts were then chosen for inclusion in the fMRI experiment, and the average rating of the chosen scripts shown in this figure (error bars representing the standard error of the mean over the 12 subjects). * denote significant matched-pair t-tests (2 tailed, p<0.01 uncorrected). Note that ratings were only compared within each rating (i.e. the three scripts were compared separately in terms of how disgusting they were, how pleasant they were and how hard they were to imagine).

**Figure S2.** Frames represent different time points of the 3 s movies depicting facial expressions of disgust, neutral and pleased gustatory experiences. See Jabbi et al., 2007 for detailed description of this part of the methods.

**Figure S3.** **Sequence of events within a single taste trial.** The person with the headphone represents an experimenter while the individual lying supine represents a participant in the scanner with three tubes protruding into a pacifier in the mouth through which various tastants are delivered. See Jabbi et al. for detailed description of this part of the methods.

**Figure S4. Structure of an imagination trial in the scanner.**
