# Supplementary figures and images for: A Common Anterior Insula Representation of Disgust Observation, Experience and Imagination Shows Divergent Functional Connectivity Pathways
Source: PLoS One. 2008 Aug 13;3(8):e2939. doi: 10.1371/journal.pone.0002939 (PMC2491556; doi:10.1371/journal.pone.0002939)

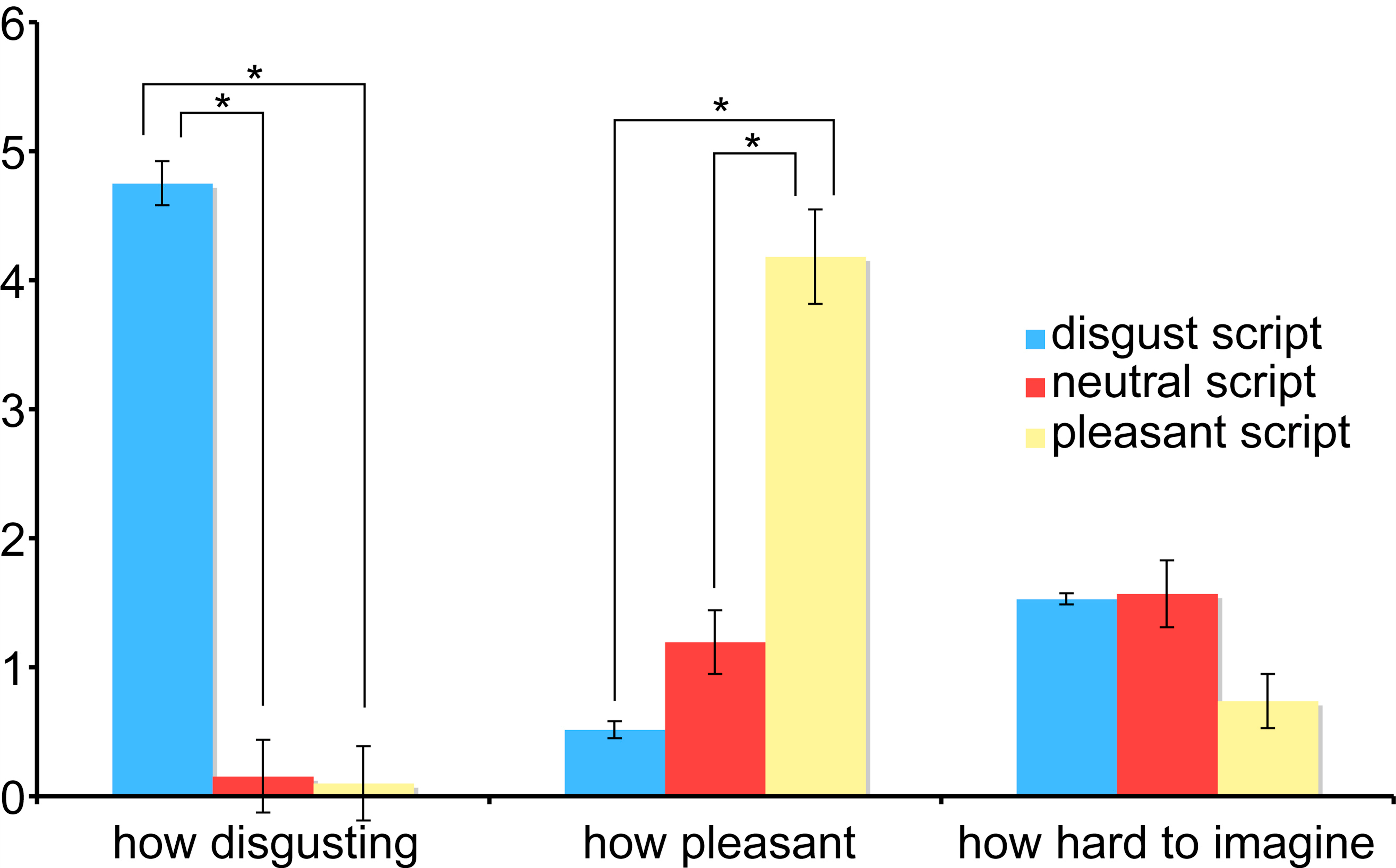

Supplement: Figure S1 — Script rating. The 12 participants of the fMRI experiment rated all 25 available scripts on a scale ranging from 0–6 according to how disgusting, how pleasant and how hard to imagine they find them. On an individual basis, the 6 most disgusting, the six most pleasant and the six most neutral (i.e. least disgusting and least pleasant) scripts were then chosen for inclusion in the fMRI experiment, and the average rating of the chosen scripts shown in this figure (error bars representing the standard error of the mean over the 12 subjects). * denote significant matched-pair t-tests (2 tailed, p<0.01 uncorrected). Note that ratings were only compared within each rating (i.e. the three scripts were compared separately in terms of how disgusting they were, how pleasant they were and how hard they were to imagine). (5.18 MB PDF) [file pone.0002939.s002.pdf]

A

B

C

Disgust

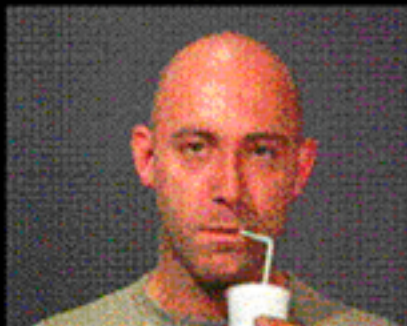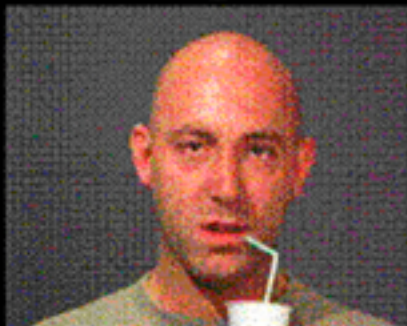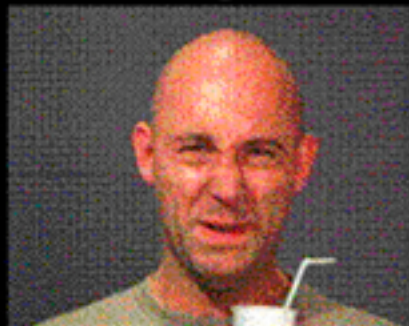

Neutral

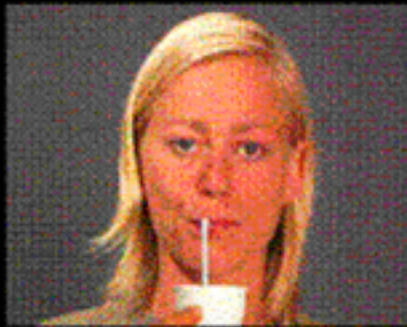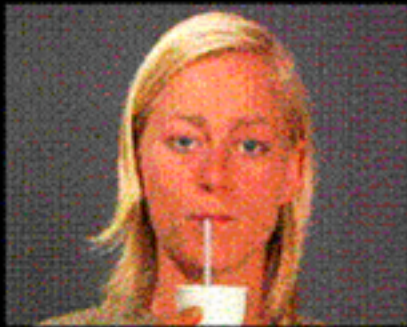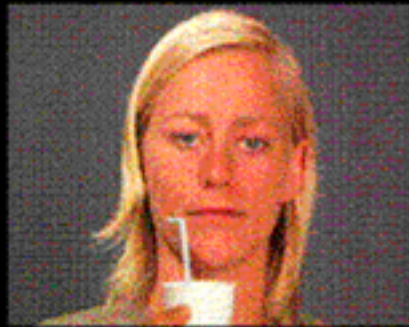

Pleasure

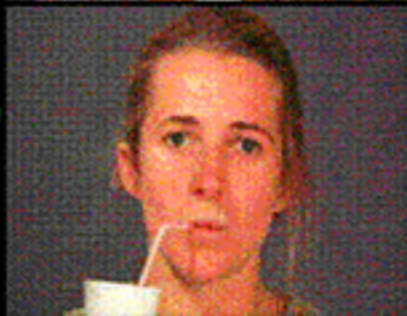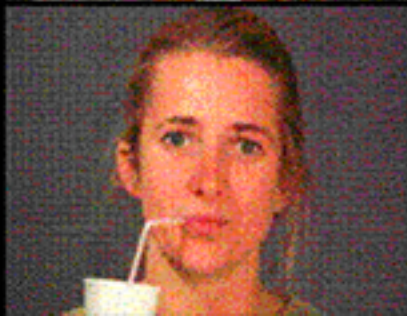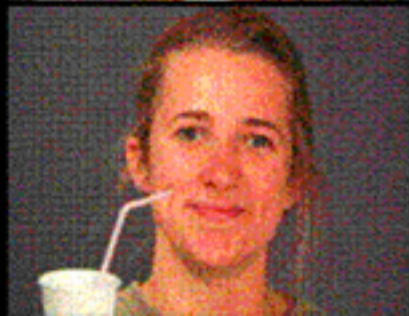

Supplement: Figure S2 — Frames represent different time points of the 3 s movies depicting facial expressions of disgust, neutral and pleased gustatory experiences. See Jabbi et al., 2007 for detailed description of this part of the methods. (1.30 MB PDF) [file pone.0002939.s003.pdf]

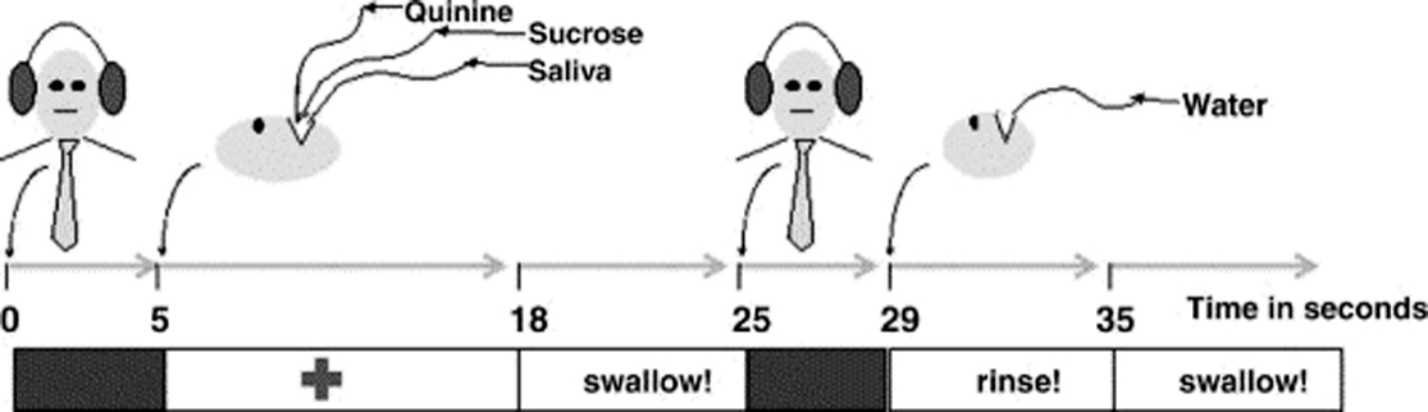

Supplement: Figure S3 — Sequence of events within a single taste trial. The person with the headphone represents an experimenter while the individual lying supine represents a participant in the scanner with three tubes protruding into a pacifier in the mouth through which various tastants are delivered. See Jabbi et al. for detailed description of this part of the methods. (0.47 MB PDF) [file pone.0002939.s004.pdf]

# Imagination trial

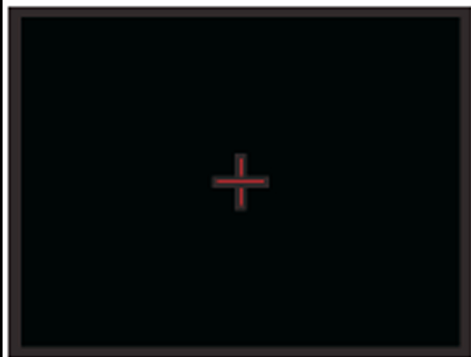

fixation 6s

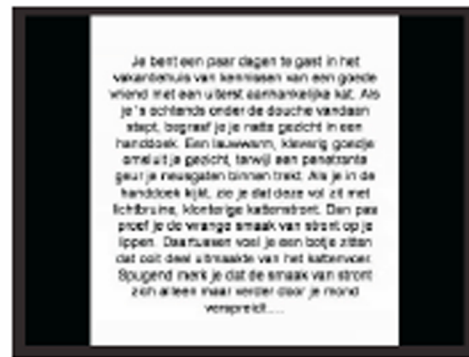

script 35s

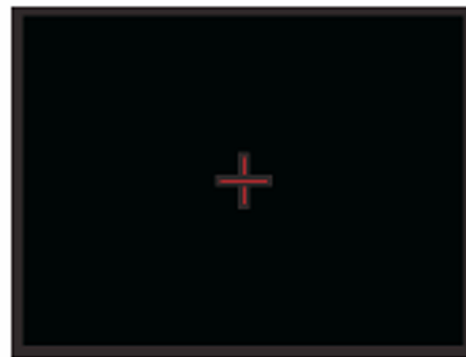

fixation 6s

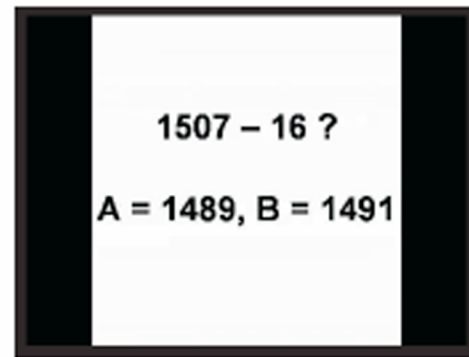

calculation 6s

Supplement: Figure S4 — Structure of an imagination trial in the scanner. (1.65 MB PDF) [file pone.0002939.s005.pdf]
