# Supplementary material for: A Common Anterior Insula Representation of Disgust Observation, Experience and Imagination Shows Divergent Functional Connectivity Pathways
Source: PLoS One. 2008 Aug 13;3(8):e2939. doi: 10.1371/journal.pone.0002939 (PMC2491556; doi:10.1371/journal.pone.0002939)
Supplement: Table S1 — (0.10 MB DOC) [file pone.0002939.s006.doc]

| **Table S1.** | |  |  |  |  |  |  | |  |  |
| --- | --- | --- | --- | --- | --- | --- | --- | --- | --- | --- |
| **Regions of Activation during the Observation, Taste and Imagination of Disgust** | | | | | | | | | |  |
| **relative to Neutral; Random effects analysis of the GLM at p < 0.001 with extent** | | | | | | | | | |  |
| **threshold at 10 voxels** | | |  |  |  |  |  |  | |  |
| **Observation: Disgust-Neutral** | | | |  |  |  |  |  | |  |
| **Region** |  |  |  | **MNI** |  |  |  |  | |  |
|  |  |  | X | Y | Z |  | voxels | t-value | | z-value |
| Insula |  |  | -31 | 11 | 5 |  | 28 | 5.72 | | 3.82 |
| Putamen |  |  | 19 | 1 | 2 |  | 33 | 7.7 | | 4.43 |
| **Taste: Disgust-Neutral** | | |  |  |  |  |  |  | |  |
| **Region** |  |  |  | **MNI** |  |  |  |  | |  |
|  |  |  | X | Y | Z |  | voxels | t-value | | z-value |
| Inferior Frontal Gyrus/Insula | | | 54 | 11 | 6 |  | 209 | 7.84 | | 4.47 |
| Bilateral Cuneus | |  | -2 | -77 | 37 |  | 167 | 6.83 | | 4.19 |
| Caudate Nucleus | |  | -15 | 8 | 9 |  | 105 | 5.67 | | 3.8 |
|  |  |  | 13 | 9 | 6 |  | 63 | 5.53 | | 3.75 |
| Putamen/Thalamus | |  | 30 | -16 | 5 |  | 41 | 7.31 | | 4.32 |
| Thalamus | |  | 6 | -27 | 2 |  | 19 | 5.99 | | 3.91 |
| Post central Gyrus | |  | -51 | -17 | 24 |  | 59 | 8.06 | | 4.52 |
| Lingual Gyrus | |  | -2 | -87 | 0 |  | 45 | 5.67 | | 3.8 |
| Anterior Cingulate Cortex | | | 3 | 31 | 15 |  | 44 | 5.4 | | 3.7 |
| Middle Frontal Gyrus | | | 37 | 42 | 20 |  | 43 | 4.95 | | 3.52 |
| Middle temporal Gyrus | | | -57 | -56 | 24 |  | 32 | 5.91 | | 3.89 |
| Superior Temporal Gyrus | | | 62 | -47 | 19 |  | 22 | 6.3 | | 4.02 |
| Superior Frontal Gyrus | | | 20 | 44 | 30 |  | 30 | 5.09 | | 3.57 |
| Supramarginal Gyrus | | | 63 | -42 | 33 |  | 16 | 5.47 | | 3.37 |
| Post Central Gyrus | |  | 50 | -10 | 35 |  | 15 | 4.77 | | 3.44 |
| Superior Frontal Gyrus | | | -20 | 55 | 17 |  | 11 | 5.22 | | 3.63 |
| Middle Cingulate Cortex | | | -2 | -38 | 46 |  | 10 | 4.46 | | 3.3 |
| Mid Cingulate/Precuneus | | | -13 | -51 | 44 |  | 10 | 4.68 | | 3.4 |
| **Imagination: Disgust-Neutral** | | | |  |  |  |  |  | |  |
| **Region** |  |  |  | **MNI** |  |  |  |  | |  |
|  |  |  | X | Y | Z |  | voxels | t-value | | z-value |
| Insula |  |  | 52 | 3 | 2 |  | 60 | 6.06 | | 3.94 |
| Superior Frontal Gyrus | | | 20 | 26 | 38 |  | 26 | 5.32 | | 3.67 |
| Inferior Frontal Gyrus | | | -46 | 4 | 9 |  | 20 | 4.91 | | 3.5 |
| Mid Temporal Gyrus | | | 49 | -40 | 9 |  | 17 | 4.97 | | 3.53 |
| DMPFC |  |  | 13 | 46 | 29 |  | 10 | 4.93 | | 3.51 |
