# Supplementary material for: A Common Anterior Insula Representation of Disgust Observation, Experience and Imagination Shows Divergent Functional Connectivity Pathways
Source: PLoS One. 2008 Aug 13;3(8):e2939. doi: 10.1371/journal.pone.0002939 (PMC2491556; doi:10.1371/journal.pone.0002939)
Supplement: Table S2 — (0.05 MB DOC) [file pone.0002939.s007.doc]

**Table S2**: Center of 5mm radius sphere used for the PPI analysis in MNI coordinates

MNI Coordinates MNI Coordinates MNI Coordinates

|  | Imagination | | | Observation | | | Taste | | |
| --- | --- | --- | --- | --- | --- | --- | --- | --- | --- |
|  | x | y | z | x | y | z | x | y | z |
| Subject 1 | 40 | 6 | 0 | 46 | 20 | -8 | 40 | 14 | -8 |
| Subject 2 | 42 | 18 | -6 | 42 | 18 | -6 | 37 | 14 | 4 |
| Subject 3 | 44 | 26 | -4 | 42 | 18 | -6 | 42 | 18 | -6 |
| Subject 4 | 42 | 14 | -8 | 42 | 12 | -8 | 44 | 20 | -4 |
| Subject 5 | 42 | 18 | -12 | 42 | 16 | -6 | 42 | 18 | -8 |
| Subject 6 | 40 | 18 | -6 | 42 | 18 | -6 | 44 | 20 | -6 |
| Subject 7 | 42 | 18 | -6 | 42 | 18 | -4 | 42 | 18 | -6 |
| Subject 8 | 42 | 18 | -6 | 38 | 14 | -6 | 48 | 16 | -2 |
| Subject 9 | 43 | 10 | -12 | 46 | 22 | -8 | 42 | 18 | -6 |
| Subject 10 | 40 | 16 | -6 | 42 | 22 | -6 | 44 | 22 | -10 |
| Subject 11 | 42 | 18 | -6 | 44 | 10 | 0 | 42 | 16 | -4 |
| Subject 12 | 42 | 18 | -10 | 42 | 18 | -8 | 42 | 18 | -6 |
| average | 41.8 | 16.5 | -6.8 | 42.5 | 17.2 | -6.0 | 42.4 | 17.7 | -5.2 |
| sem | 0.4 | 1.4 | 1.0 | 0.6 | 1.1 | 0.7 | 0.8 | 0.7 | 1.0 |
